# Risk Factors Associated with Mortality among Patients with COVID-19: Analysis of a Cohort of 1213 Patients in a Tertiary Healthcare Center

**DOI:** 10.3390/jcm11102780

**Published:** 2022-05-14

**Authors:** Carlos Alfonso Romero-Gameros, Guadalupe Vargas-Ortega, Mario Enrique Rendón-Macias, Carlos Fredy Cuevas-García, Tania Colín-Martínez, Luis Alejandro Sánchez-Hurtado, Lourdes Josefina Balcázar-Hernández, Iván Emilio De la Cruz-Rodríguez, Enid Karina Pérez-Dionisio, Perla Michelle Retana-Torres, Elsy Sarahí García-Montesinos, Mayra Alejandra López-Moreno, Marielle Intriago-Alor, Salomón Waizel-Haiat, Baldomero González-Virla

**Affiliations:** 1 Otorhinolaryngology Service, Hospital de Especialidades, Centro Médico Nacional Siglo XXI, Instituto Mexicano del Seguro Social, Mexico City 06720, Mexico; carlos.romero.gameros@gmail.com (C.A.R.-G.); malm92.22@gmail.com (M.A.L.-M.); mariellealor@gmail.com (M.I.-A.); swaizel@hotmail.com (S.W.-H.); 2 Endocrinology Service, Hospital de Especialidades, Centro Médico Nacional Siglo XXI, Instituto Mexicano del Seguro Social, Mexico City 06720, Mexico; ludab_2@hotmail.com (L.J.B.-H.); i.emilio.delacruz@gmail.com (I.E.D.l.C.-R.); enid_karina@hotmail.com (E.K.P.-D.); perlamret@gmail.com (P.M.R.-T.); sarahi.montesinos@gmail.com (E.S.G.-M.); 3 Department of Biostatistics, Faculty of Health Sciences, Universidad Panamericana, Mexico City 03920, Mexico; drmariorendon@gmail.com; 4 Neurology Service, Hospital de Especialidades, Centro Médico Nacional Siglo XXI, Instituto Mexicano del Seguro Social, Mexico City 06720, Mexico; carlos.cuevasg@imss.gob.mx; 5 Emergency Service, Hospital de Especialidades, Centro Médico Nacional Siglo XXI, Instituto Mexicano del Seguro Social, Mexico City 06720, Mexico; tania.colin2a@gmail.com; 6 Critical Care Medicine Service, Hospital de Especialidades, Centro Médico Nacional Siglo XXI, Instituto Mexicano del Seguro Social, Mexico City 06720, Mexico; luis.sanchezhur@imss.gob.mx

**Keywords:** COVID-19, mortality, prognostic factors, SARS-CoV-2

## Abstract

The presence of cardio-metabolic and respiratory comorbidities, immunosuppression, and chronic kidney disease have been associated with an increase in mortality from COVID-19. The objective of this study is to establish the risk factors associated with 30-day mortality in a cohort of hospitalized patients with COVID-19. This paper conducts a retrospective and analytical study of patients hospitalized for COVID-19 in a tertiary care center. A Cox proportional hazard analysis was performed to estimate the association of comorbidities with 30-day mortality. A total of 1215 patients with a median age of 59 years were included. In the adjusted Cox proportional hazards regression model, hypothyroidism, D-dimer ≥ 0.8 μg/mL, LHD ≥ 430 IU/L, CRP ≥ 4.83 ng/mL, and triglycerides ≥ 214 mg/dL were associated with an increased risk of death. The presence of a history of hypothyroidism and biomarkers (D-dimer, lactic dehydrogenase, CRP, and triglycerides) were associated with an increase in mortality in the studied cohort.

## 1. Introduction

The COVID-19 pandemic, caused by the SARS-CoV-2 virus infection, began in the Wuhan region of China in late 2019 and reached Mexico in February 2020, with a frenzied increase during the following months. The SARS-CoV-2 infection was mainly characterized by acute respiratory symptoms and related systemic complications. The presence of cardio-metabolic (diabetes, hypertension, and obesity) and respiratory (asthma and chronic obstructive pulmonary disease) comorbidities, immunosuppression, and chronic kidney disease (CKD) were all aggravating factors in the evolution and possible triggers for increased mortality in Mexico [1,2,3,4,5].

Bello-Chavolla et al. conducted a study within a Mexican sample where they investigated the specific risk factors associated with mortality, in particular the impact of diabetes and obesity on lethality in COVID-19 patients. Their results showed a higher risk of death after 30 days in patients with the following description: over 65 years old, diabetes mellitus 2 (DM2), obesity, CKD, chronic obstructive pulmonary disease (COPD), immunosuppression, and hypertension [3].

At the time of writing this manuscript (May 6th, 2022), 516,326,823 cases have been recorded worldwide, with a total of 6,248,434 deaths. In Mexico, the figure has reached 5,739,680 cases and 324,334 deaths, representing a case fatality rate of 1.21% and 5.65%, respectively [6]. This fatality rate could be higher amongst high-risk populations with a greater number of comorbidities. Although there are numerous reports in the Mexican population and in the world on various clinical and biochemical risk factors associated with the risk of mortality in patients with SARS-CoV-2 infection, a high-risk analysis will allow us to understand this phenomenon with greater precision.

Thus, the aim of this study is to establish the risk factors (clinical characteristics, comorbidities, and other predictors) associated with a 30-day mortality in a cohort of patients hospitalized due to COVID-19 in a tertiary care center where the prevalence of comorbidities was more pronounced.

## 2. Materials and Methods

### 2.1. Study Design and Patient Population

An observational, retrospective, and analytical study was conducted among COVID-19 patients at the Hospital de Especialidades del Centro Médico Nacional Siglo XXI del Instituto Mexicano del Seguro Social (a tertiary center) in Mexico City, Mexico, during the period between 1 March 2020 and 30 May 2021. Patients were included using the following criteria: ≥18 years old, with a COVID-19 diagnosis confirmed by RT–PCR, of any gender, and with complete labs for the purposes of the study. The primary outcome was death from COVID-19 and its complications within 30 days of hospital admission. The death certificates of 243 patients of the studied cohort were obtained.

The present study was approved by the local ethics and research committee (Registry identifier: R-2020-3601-245) and was consistent with the ethical guidelines of the 1975 Helsinki Declaration and the Mexican General Health Law on Research for Health Studies.

### 2.2. Data Collection

Data were collected retrospectively through consultation of the electronic clinical records. The demographic data, comorbidities, symptomatology at admission, laboratory results, and therapies used were all collected.

### 2.3. COVID-19 Diagnosis

The identification of SARS-CoV-2 was obtained by real-time RT–PCR in nasopharyngeal exudate samples processed at the Central Epidemiology Laboratory of the National Medical Center “La Raza” of the Mexican Social Security Institute, following international standards for infectious substances.

### 2.4. Definition of Variables

Co-morbidities were defined as follows: a chronic obstructive pulmonary disease (COPD) diagnosis of a postbronchodilator FEV1/FVC ratio around <0.70 [7]; asthma according to the Global Initiative for Asthma 2020 criteria [8]; heart disease as a diagnosis of coronary artery disease, congestive heart failure, or heart rhythm problems; CKD as a glomerular filtration rate below 60 mL/min [9]; immunodeficiency disease as the diagnosis of any immunodeficiency disorder, whether primary or secondary; diabetes as a fasting plasma glucose (FPG) level of 126 mg/dL or higher, a 2 h plasma glucose level of 200 mg/dL or higher during a 75-g oral glucose tolerance test, or a hemoglobin A1c level of 6.5% or higher [10]; hypertension as systolic blood pressure ≥140 mm Hg and/or diastolic blood pressure ≥90 mm Hg [11]; and primary hypothyroidism as a TSH level > 4.2 µIU/mL and T4 level < 0.93 ng/dL, and in the case of central hypothyroidism, a T4 level < 0.8 ng/dL.

Analysis for detecting serum glucose, d-dimer, lactic dehydrogenase, ferritin, c-reactive protein, and triglycerides were performed in the laboratory of the Hospital de Especialidades Centro Médico Nacional Siglo XXI. Samples for laboratory analysis were obtained at the time of hospital admission.

The treatments used at the hospital were classified as: immunomodulatory therapy (systemic corticosteroids or tocilizumab), antimicrobial therapy (macrolide, quinolones, beta-lactam, hydroxychloroquine, azithromycin, or lopinavir/ritonavir), or anticoagulant therapy (low-molecular-weight heparin).

### 2.5. Statistical Methods

Descriptive and inferential statistics were used for data analysis, taking into account measures of central trends and dispersion. The chi-square test was used to compare frequencies and proportions. The Mann–Whitney U test or Student’s *t* test was used to compare quantitative variables. The Shapiro–Wilk test was used to determine the normality of the distribution of the variables. A receiver operating characteristic (ROC) analysis was performed to determine the best cutoff points for the following continuous quantitative variables: age, D-dimer, LDH, ferritin, CRP, and triglycerides. To determine the best cut-off points, sensitivity, specificity, accuracy, and likelihood ratio (LR)+ and LR– were taken into account.

A Cox proportional hazard analysis was performed to estimate the magnitude of the relationship between the different COVID-19-associated comorbidities (and biochemical parameters) and mortality during the 30 days after admission. The variables included in the regression model were made taking into account biological plausibility and statistical significance. A Kaplan–Meier plot was used to estimate the probability of survival at 30 days after hospitalization, while the log-rank test was used to compare the difference in survival probability for different groups of COVID-19 patients.

A two-sided *p* value was used for the in-between group difference with respect to the primary outcome. A *p* value of *p* < 0.05 was considered statistically significant. The statistical software used was SPSS version 25.0 (IBM SPSS Statistics for Windows, IBM Corp, Armonk, NY, USA), Stata SE software version 16 (StataCorp, College Station, TX, USA), and GraphPad Prism version 8.0 for Windows (GraphPad Software, San Diego, CA, USA).

## 3. Results

**Figure 1:**
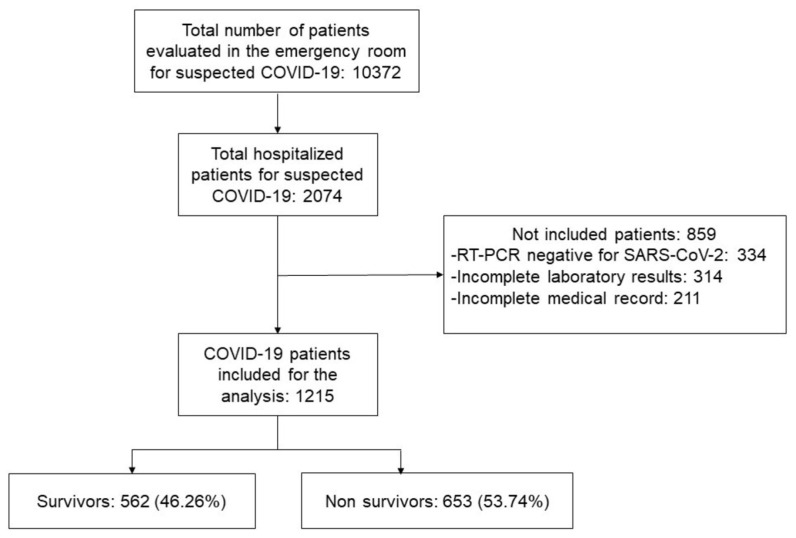
Study flow diagram.

During the study period, 2074 patients were hospitalized with suspected COVID-19, out of whom 1215 met our criteria for mortality risk analysis (Figure 1). The number of deaths recorded was 653 (53.7%).

### 3.1. Baseline Characteristics

The median age for the total study population was 59 years (IQR, 47–69 years). The median age in the group of patients who died was 63 years (IQR, 52–72 years) vs. 54 years (IQR, 43–66 years), with a difference of 9 years (95% CI; 6–11 years) between the two groups. Of the total group, 764 patients (62.8%) were male, with similar proportions between non-survivors (NS) and survivors (Table 1).

In the group of patients studied, the presence of fever and dyspnea was significantly more frequent than in the group of non-survivors (Table 1). Regarding biomarkers, statistically significant differences were observed in the concentrations of fasting plasma glucose, D-dimer, LDH, ferritin, CRP, and triglycerides when comparing both groups (see Table 1). Similarly, a higher proportion of type 2 diabetes (T2D), hypertension, liver disease, and hypothyroidism was observed in the non-survivor group, reaching statistical significance (Table 1). The proportion of patients with ≥2 comorbidities was significantly higher in the non-survivor group than in the survivor group. (See Table 1).

### 3.2. ROC Analysis to Determine Cut-Off Points

The cut-off points of the different continuous quantitative variables and their respective sensitivity, specificity, accuracy, LR+, and LR−, are presented below: D-dimer ≥ 0.8 μg/mL, sensitivity 85.30%, specificity 40.93%, accuracy 64.77%, LR+ 1.4439, and LR− 0.3592; LDH ≥ 430 IU/L, sensitivity 63.71%, specificity 62.28%, accuracy 63.05%, LR+ 1.68, and LR− 0.58; Ferritin ≥ 413.5 ng/mL, sensitivity 93.72%, specificity 18.86%, accuracy 59.09%, LR+ 0.15, and LR− 0.33; CRP ≥ 4.83 mg/dL, sensitivity 85.3%, specificity 33.99%, correctly classified 61.56%, LR+ 1.29, and LR− 0.43; Triglycerides ≥ 214 mg/dL, sensitivity 58.04%, specificity 62.28%, correctly classified 60%, LR+ 1.53, and LR− 0.67.

### 3.3. Cox Proportional Hazards and Kaplan–Meier Analysis

**Figure 2:**
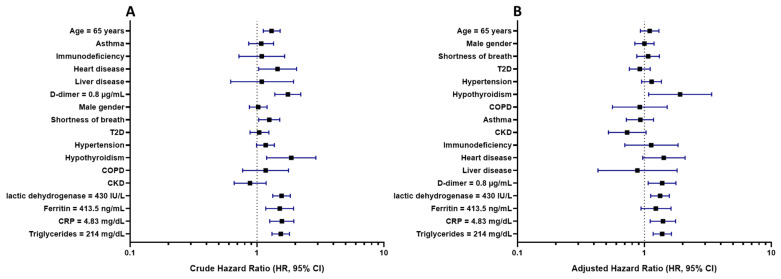
Cox proportional hazard models for 30-day mortality in hospitalized patients with COVID-19 (n = 1215). Abbreviations: LDH, lactic dehydrogenase; T2D, type 2 diabetes; CRP, C-reactive protein; COPD, chronic pulmonary disease; and CKD, chronic kidney disease. (**A**) Univariate Cox proportional hazards regression model. (**B**) Multivariable Cox proportional hazards regression model.

In the adjusted Cox proportional hazards regression model, hypothyroidism, D-dimer ≥ 0.8 μg/mL, LDH ≥ 430 IU/L, CRP ≥ 4.83 ng/mL, and triglycerides ≥ 214 mg/dL were significantly associated with an increased risk of death (Table 2, Figure 2). For the Cox proportional hazards model, a chi2 LR of 91.74 was obtained with a *p* value < 0.001.

Similarly, a crude HR of 0.65 (95% CI [0.48–0.88], *p* = 0.007) was found for the age group of <40 years old (Table 2, Figure 2).

**Figure 3:**
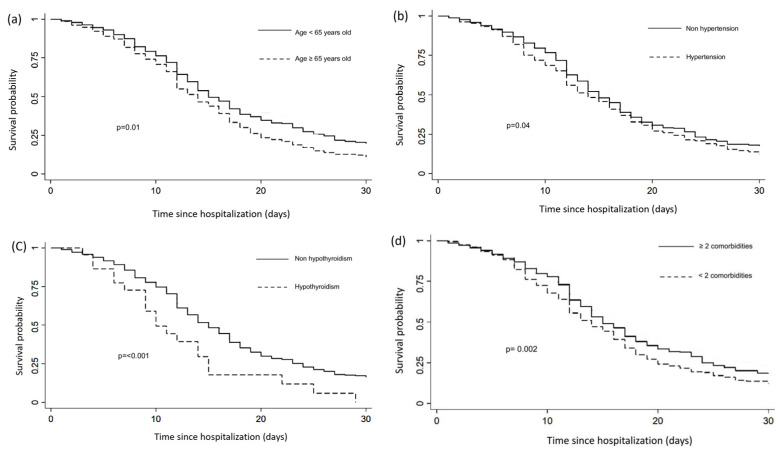
Thirty-day survival of hospitalized COVID-19 patients after hospital admission, stratified by age ≥ 65 years (**a**) hypertension, (**b**) hypothyroidism, (**c**) ≥2 comorbidities, and (**d**) (n = 1215).

**Figure 4:**
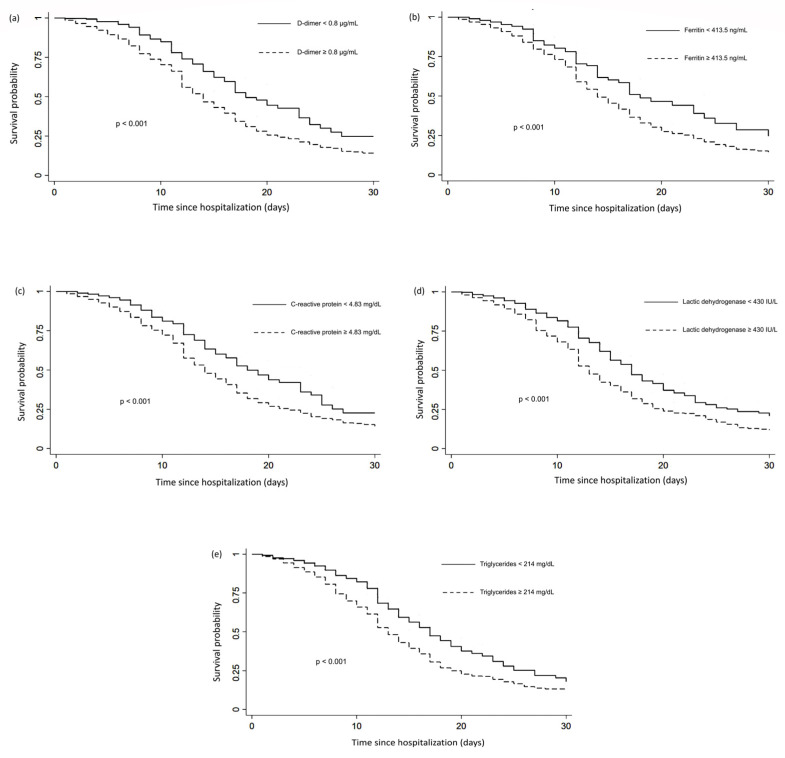
Thirty-day survival of hospitalized COVID-19 patients after hospital admission stratified by D-dimer ≥ 0.8 μg/mL, (**a**) ferritin ≥ 413.5 ng/mL, (**b**) C-reactive protein ≥ 4.83 mg/dL, (**c**) lactic dehydrogenase ≥ 430 IU/L, (**d**) triglycerides ≥ 214 mg/dL, and (**e**) (n = 1215).

The Kaplan–Meier analysis showed that the probability of 30-day survival in hospitalized patients ≥65 years was 11% vs. 20% in those <65 years; survival in patients with hypertension was 12% vs. 18% in those without hypertension; and survival in patients with ≥two comorbidities was 12% vs. 19% in patients with <two comorbidities (Figure 3). A median survival of 10 days was observed for hypothyroid patients compared with 15 days for non-hypothyroid patients (Figure 3). Regarding biomarkers, the following survival estimates were obtained: d-dimer D ≥ 0.8 μg/mL was 13% vs. 25% d-dimer D < 0.8 μg/mL; ferritin ≥ 413.5 ng/mL 14% vs. 25% < 413.5 ng/mL; CRP ≥ 4.83 ng/mL 14% vs. 23% CRP < 4.83 ng/mL; triglycerides ≥ 214 mg/dL 13% vs 17% < 214 mg/dL; and LDH ≥ 430 IU/L 12% vs. 21% LDH < 430 IU/L (Figure 4).

## 4. Discussion

For the present study, we present the analysis of a group of 1215 adult patients hospitalized with COVID-19. The objective was to establish which risk factors were associated with a 30-day mortality. We anticipated that the contribution would be to explore the possible additive effect of an initial SARS-CoV-2 infection, plus the presence of comorbidities and biomarkers on 30-day mortality. In the Cox proportional hazard model, a history of hypothyroidism and biomarkers (D-dimer ≥ 0.8 μg/mL, lactic dehydrogenase ≥ 430 IU/L, CRP ≥ 4.83mg/dL, and triglycerides ≥ 214 mg/dL) were significantly associated with increased mortality.

As in the study by Bello-Chavolla et al., ages over 65 increased the risk of death [HR 2.02, 95%CI (1.89–2.16), *p* < 0.001] [3]; in our study, we obtained an HR of 1.30 (95% CI [1.12–1.52], *p* = 0.001). Megan O’Driscoll et al. estimated that the fatality rate due to SARS-CoV-2 infection showed a logarithmic linear increase related to age after 30 years-old [12] and Escobedo-de la Peña et al. also found a case fatality rate (CFR) that increased with age; more than half of the deceased subjects were ≥70 years-old [5]. It has been acknowledged that aging affects the function of the adaptive and innate immune system, and therefore increases susceptibility to infections, and suppressed Natural Killer (NK) cell cytolytic activity has been observed more in elderly patients compared to younger subjects. NK cells are a family of innate immune cells that play an essential role in antiviral immunity [13].

Hypertension, like other cardiovascular diseases, has been associated with increased mortality in patients hospitalized for COVID-19 [4,14,15]. In our study, it was the most frequent comorbidity in the NS group (34.46%). However, in the multivariate model, no significant association was found with mortality in the cohort of patients studied. Animal models suggest that RAAS blockers may increase ACE2 expression and potentially increase the risk of SARS-CoV-2 infection. On the other hand, a recent meta-analysis published by Chang Chu et al. concluded that angiotensin-converting enzyme inhibitors (ACEIs) reduce the risk of SARS-CoV-2 infection and all causes of COVID-19 mortality, including the risk of non-COVID-19 pneumonia [16]. The exact mechanism of hypertension related to the COVID-19 severity remains unclear [15].

T2D was the second most frequent comorbidity in the NS group (28.02%). However, no significant association was found with mortality. Our findings contrast with those reported in other studies such as the one by Muhammad M AbdelGhaffar et al. who reported an OR for T2D mortality of 1.58 (95% CI 1.14–2.19; *p* = 0.006 [17]. Lana pinto et al. performed a meta-analysis where the pooled OR was 3.53 (95% CI; 1.48–8.39) [18], similar to the Zeng-Hong Wu et al. OR of 1.75 (95% CI 1.31–2.36; *p* = 0.0002) [19]. The impaired immune system coupled with the metabolic imbalance observed in patients with T2D increases susceptibility to infection and perhaps a more severe disease. NK cells are innate lymphocytes that detect and destroy virus-infected cells. It has been observed that there is an increase in the number of dysfunctional NK cells in patients with T2D, probably secondary to an increase in reactive oxygen species. Hyperglycemia itself alters the quaternary structure of proteins in NK cells, inducing their apoptosis and decreasing their viral clearance capacity [20]. This possibly explains the increased risk of death for patients with T2D.

A history of primary hypothyroidism was associated with an increased risk of mortality, with an HR of 1.91 (95% CI; 1.08–3.39, *p* = 0.02), and as in the general population, it was more frequent in the female gender compared to males [21], both in the total group (68.8%, *p* = 0.002) and in the NS group (65%, *p* = 0.005). The relationship between hypothyroidism and mortality in COVID-19 is unclear. Maaike van Gerwen et al. found no increased risk of hospitalization (OR 1.23, 95% CI [0.88–1.70]), mechanical ventilation (OR 1.17, 95% CI [0.81–1.69]), or death (OR 1.07, 95% CI [0.75–1.54]) for patients with hypothyroidism [22]. However, Fachreza Aryo Damara et al. in their systematic review with meta-analysis in 2021 found that having unspecified thyroid disease was associated with poor COVID-19 outcomes (including mortality) (RR 1.91, 95% CI [1.38–2.65]) and hypothyroidism (RR 1.90, 95% CI [1.42–2.55]) [23]. It has been shown that thyroid hormones can affect the production of NK cells [13]. Treatment with triiodothyroxine (T3) can induce IL-2 receptor expression on peripheral blood mononuclear cells; administration of low doses of thyroxine enhances the stimulatory effect of interferon on NK cells, such that there is evidence of an interaction between thyroid function and NK cell activity [13]. Based on the above, a history of hypothyroidism could influence the deterioration of the cellular immune response to SARS-CoV-2 infection, increasing the probability of death in hospitalized patients.

Regarding the association between triglyceride concentration and mortality, our analysis yielded an increased risk when its concentration was ≥214 mg/dL (HR of 1.38 (95% CI [1.17–1.63], *p* < 0.001). Wen Dai et al. had already found this association in their study of deceased patients who had higher levels than survivors (179 vs. 134 mg/dL, *p* < 0.001), with an OR for mortality of 2.3 (95% CI [1.4–3.7], *p* = 0.001) [24]. Previous studies have shown that infection and inflammation induce hypertriglyceridemia due to inhibition of serum clearance by lipoprotein lipase, a key enzyme in triglyceride catabolism. On the other hand, recent studies suggest a promoting effect of triglycerides in inflammation; high levels favor macrophage extravasation to tissues. This evidence may suggest a detrimental effect of hypertriglyceridemia on leukocyte activation in patients with COVID-19, placing them at high risk for severe disease [24].

The identification of biomarkers associated with mortality measured early in hospitalized patients with COVID-19 could be a useful diagnostic tool for making therapeutic decisions in high-risk patients. In fact, we found a significant increase in the risk of mortality in those patients with D-dimer concentrations ≥ 0.8 μg/mL (HR 1.38, 95% CI [1.07–1.37], *p* = 0.01), LDH ≥ 430 IU/L (HR 1.33, 95% CI [1.12–1.57], *p* = 0.001), CRP ≥ 4.83mg/dL (HR 1.40, 95% CI [1.11–1.76], *p* = 0.004). With respect to a ferritin concentration ≥413.5 ng/mL, there was a trend toward higher risk as this increased HR 1.23 (95% CI; 0.94–1.62, *p* = 0.12). In a meta-analysis and systematic review published by Preeti Malik et al. where they evaluated laboratory findings and their relationship with outcomes in patients hospitalized for COVID-19, they found that biomarkers such as: elevated D-dimer (pooled-OR 3.39; 95% CI [2.66–4.33], *p* < 0.00001), LDH (pooled-OR 5.48; 95% CI [3.89–7.71], *p* < 0.00001), and elevated C-reactive protein (pooled-OR 4.37; 95% CI [3.37–5.68], *p* < 0.001) were markers independently associated with a high risk of poor outcome. Likewise, other authors have also described the association of CPR, LDH, D-dimer, and ferritin biomarkers [2,25,26,27]. Biomarkers (CPR, D-dimer, LDH, and ferritin) are quantitative measurements that reflect the pathophysiology of the disease and help the clinician recognize the severity of the condition. They help guide therapeutic decisions to improve patient prognosis [28]. CRP is an acute-phase protein synthesized in hepatocytes in response to IL-6; its concentrations are elevated in different inflammatory processes including infectious ones and are therefore useful in the diagnosis and analysis of the severity of the infectious process [28,29]. Elevated D-dimer concentrations, also associated with C-reactive protein levels, have been associated with a poor prognosis in patients with COVID-19 [17], an effect associated with hypoxia due to severe pneumonia and increased inflammatory response, conditions related to a state of hypercoagulability, resulting in disseminated intravascular coagulation and multiorgan failure [17].

Increased serum LDH levels are a marker of the presence of tissue injury, necrosis, and hypoxia and is an independent marker associated with increased mortality in patients with sepsis [30]. In a study by Yi Han et al., LDH was found to be a robust predictor for early recognition of lung injury and its severity due to COVID-19 [31]. In our study, it was also found to be an unfavorable prognostic factor for survival.

The exact role of ferritin in the pathophysiology of COVID-19 has not yet been fully established. However, what is currently known is that, in response to tissue injury, cytokines stimulate the production of defense proteins by the liver, including C-reactive protein and ferritin. Transcription and translation of ferritin are induced by IL-1β, IL-6, and IFN-γ. Additionally, macrophages and damaged cells account for elevated ferritin values. Ferritin promotes the release of proinflammatory mediators and increases the inflammatory burden, resulting in a vicious circle. Ferritin achieves this by activation of NF-ԟB, leading to a positive regulation of ferritin gene transcription [32]. In our study, its impact appears not to have been very significant on the risk of death.

The strengths of our study include having an acceptable sample size, which allows us to accurately estimate which covariates were associated with mortality in a group of hospitalized patients with COVID-19. The cohort of hospitalized patients was adequately characterized and followed-up on for the established time. The limitations of the study are its retrospective nature; the inclusion of severe cases referred to us by a third level center, which could limit the external validity of the study; the difficulty in obtaining all the death certificates, which would allow corroborating the final cause of death; and the presence of a probable differential bias, since the analysis of the patients’ information was done according to the background info and not the morbidity control. Finally, it was not possible to evaluate the weight and body mass index of the patients, due to underreporting in the clinical record.

## 5. Conclusions

The data obtained in our study suggest that the presence of a history of hypothyroidism, a D-dimer ≥ 0.8 μg/mL, lactic dehydrogenase ≥ 430 IU/L, CRP ≥ 4.83 mg/dL, and triglycerides ≥ 214 mg/dL were associated with an increase in mortality in the studied cohort. A significant number of comorbidities probably influence mortality as a constant risk.

## Figures and Tables

**Table 1:** Baseline characteristics of 1215 hospitalized patients with a diagnosis of COVID-19.

Variable	Non-Survivors (n = 653)	Survivors (n = 562)	Differences (95% CI) *	*p*
Age, median years, mean ± SD	61.21 ± 14.23	54.13 ± 15.09	7.08 (8.73 to 5.42)	<0.001 ^a^
Gender, n (%)				0.15 ^b^
Women	230 (35.22)	221 (39.32)	−4.1 (−9.5 to 1.3)	
Men	423 (64.78)	341 (60.68)	4.1 (−1.3 to 9.5)	
Symptoms, n (%)				
Fever	452 (69.33)	348 (61.92)	7.41 (2.05 to 12.76)	0.007 ^b^
Odynophagia	219 (33.59)	234 (41.64)	−8.05 (−13.50 to −2.59)	0.004 ^b^
Chest pain	263 (40.34)	266 (47.33)	−6.99 (−12.57 to −1.40)	0.01 ^b^
Asthenia	425 (65.18)	354 (62.99)	2.19 (−3.22 to 7.60)	0.42 ^b^
Myalgia	341 (52.3)	313 (55.69)	−3.39 (−9.00 to 2.22)	0.23 ^b^
Headache	273 (41.87)	262 (46.62)	−4.75 (−10.34 to 0.84)	0.09 ^b^
Rhinorrhea	130 (19.94)	141 (25.09)	−5.15 (−9.86 to −0.43)	0.03 ^b^
Anosmia	112 (17.18)	131 (23.1)	−5.92 (−10.44 to −1.39)	0.008 ^b^
Shortness of breath	520 (79.75)	385 (68.51)	11.24 (6.31 to 16.16)	<0.001 ^b^
Comorbidities, n (%)				
COPD	23 (3.53)	12 (2.14)	1.39 (−0.46 to 3.24)	0.14 ^b^
Asthma	87 (13.32)	73 (12.99)	0.33 (−3.48 to 4.14)	0.86 ^b^
T2D	183 (28.02)	112 (19.93)	8.09 (3.31 to 12.86)	<0.001 ^b^
Hypertension	225 (34.46)	124 (22.06)	12.4 (7.39 to 17.40)	<0.001 ^b^
Heart disease	35 (5.36)	20 (3.56)	1.8 (−0.50 to 4.10)	0.13 ^b^
Nephropathy	48 (7.35)	40 (7.12)	0.23 (−2.68 to 3.14)	0.87 ^b^
Immunodeficiency	23 (3.52)	20 (3.56)	−0.04 (−2.12 to −2.04)	0.79 ^b^
Liver disease	12 (1.84)	3 (0.53)	1.31 (0.11 to 2.50)	0.04 ^b^
Hypothyroidism	20 (3.06)	2 (0.36)	2.7 (1.28 to 4.11)	<0.001 ^b^
≥2 comorbidities	276 (42.27)	129 (22.95)	19.32 (14.17–24.4)	<0.001 ^b^
Biochemical markers				
Fasting plasma glucose, mg/dL median (IQR)	135 (106–206)	113 (95–162)	22 (13 to 30)	<0.001 ^c^
D-dimer, μg/mL median (IQR)	2.07 (1.05–5.97)	1.01 (0.56–2.54)	1.06 (0.75 to 1.36)	<0.001 ^c^
Lactic dehydrogenase, median IU/L (IQR)	512 (362–655)	374.5 (278–503.5)	137.5 (110 to 161)	<0.001 ^c^
Ferritin, ng/mL median (IQR)	1275 (709.1–1622)	977.7 (448–1395.94)	297.3 (174.64 to 419.55)	<0.001 ^c^
C-reactive protein, mg/dL median (IQR)	14.2 (7.58–21.82)	9.49 (2.63–14.2)	4.71(3.48 to 5.83)	<0.001 ^c^
Triglycerides, mg/dL median (IQR)	226 (163–277)	190.5 (136–226)	35.5 (27 to 43)	<0.001 ^c^

Abbreviations: SD, standard deviation; IQR, interquartile range; COPD, chronic obstructive pulmonary disease; T2D, type 2 diabetes; and SAH, systemic arterial hypertension. * For continuous variables, the difference in medians and means is shown. For categorical variables, the absolute difference in percentage points is shown. ^a^ *p* value estimated by Student’s *t* test for independent samples test between group of non-survivors and survivors. ^b^ *p* value estimated by Pearson’s Xi^2^ test between the group of non-survivors and the survivors. ^c^ *p* value estimated by Mann–Whitney U test between the group of non-survivors and the survivors.

**Table 2:** Cox proportional hazard model for 30-day mortality in hospitalized patients with COVID-19 (n = 1215).

Variable	Crude HR	95% CI	*p*	Adjusted HR *	95% CI	*p*
Age < 40 years	0.65	0.48–0.88	0.007	-	-	-
Age ≥ 65 years	1.30	1.12–1.52	0.001	1.1	0.93–1.30	0.22
Male gender	1.02	0.87–1.20	0.74	1	0.84–1.19	0.97
Shortness of breath	1.25	1.03–1.51	0.021	1.07	0.87–1.31	0.4
T2D	1.04	0.88–1.24	0.58	0.92	0.76–1.11	0.42
Hypertension	1.17	0.99–1.37	0.52	1.14	0.95–1.36	0.14
Hypothyroidism	1.86	1.19–2.91	0.006	1.91	1.08–3.39	0.02
COPD	1.17	0.77–1.77	0.45	0.92	0.56–1.51	0.74
Asthma	1.08	0.86–1.35	0.48	0.93	0.72–1.18	0.56
CKD	0.88	0.66–1.18	0.41	0.73	0.52–1.03	0.7
Immunodeficiency	1.09	0.72–1.65	0.67	1.13	0.7–1.84	0.6
Heart disease	1.45	1.03–2.05	0.03	1.42	0.97–2.09	0.06
Liver disease	1.09	0.62–1.94	0.74	0.88	0.43–1.81	0.74
D–dimer ≥ 0.8 μg/mL	1.75	1.38–2.21	<0.001	1.38	1.07–1.77	0.01
Lactic dehydrogenase ≥ 430 IU/L	1.56	1.33–1.83	<0.001	1.33	1.12–1.57	0.001
Ferritin ≥ 413.5 ng/mL	1.51	1.17–1.94	0.001	1.23	0.94–1.62	0.12
CRP ≥ 4.83 mg/dL	1.57	1.26–1.95	<0.001	1.40	1.11–1.76	0.004
Triglycerides ≥ 214 mg/dL	1.54	1.31–1.80	<0.001	1.38	1.17–1.63	<0.001

Abbreviations: T2D, type 2 diabetes; COPD, chronic pulmonary disease; CKD, chronic kidney disease; CRP, C-reactive protein; and 95% CI, 95% confidence interval.* Cox proportional hazard model including hypertension, T2D, gender, hypothyroidism, COPD, asthma, CKD, Immunodeficiency, heart disease, liver disease, D dimer ≥ 0.8 μg/mL, lactic dehydrogenase ≥ 430 IU/L, ferritin ≥ 413.5 ng/mL, CRP ≥ 4.83 mg/dL, and triglycerides ≥ 214 mg/dL.

## Data Availability

The datasets used and/or analyzed during the current study are available from the corresponding author on reasonable request.
